# Immunosuppressive therapy in dermatology and PML

**DOI:** 10.1111/j.1610-0387.2008.06993.x

**Published:** 2009-01

**Authors:** Wolfram Sterry, Martine Bagot, Carlos Ferrandiz, Knud Kragballe, Kim Papp, Georg Stingl

Progressive multifocal leukoencephalopathy (PML) is a rare, demyelinating disease of the central nervous system that usually leads to death or severe disability. Affected patients present with signs and symptoms including impaired cognition, visual disturbances, hemiparesis, altered mental state and behavioural changes. PML is caused by activation of the John Cunningham (JC) virus. JC virus resides in latent form in up to 80 percent of adults. Reactivation of JC virus occurs in immunocompromised patients. The factors leading to activation of the latent infection are not fully understood. The diagnosis of PML is established based on a detailed medical history, physical examination, suggestive MRI findings and the presence of the JC virus detected in the cerebrospinal fluid by PCR. PML has been reported in the published literature in HIV-positive patients, as well as in patients receiving immunosuppressive agents because of organ transplantation, cancer or autoimmune diseases. PML has been reported in patients receiving classical immunosuppressive drugs or biologics or both [1]. There are no known interventions that can prevent or treat PML successfully.

Recently, two confirmed cases of PML have been reported in US patients receiving efalizumab (Raptiva®) for chronic plaque psoriasis [2]. One male patient (age 70) and one female patient (age 73) had received efalizumab for 4 and 3.8 years, respectively. These are the first confirmed cases of PML in psoriasis patients receiving immunosuppressive therapy. Today, the global exposure of patients on efalizumab is 47,000 patient-years.

Until more information is available and further guidance is received from the health authorities, a group of independent expert dermatologists advise to strictly follow the Summary of Product Characteristics of efalizumab [3]. Dermatologists should carefully weigh risks and benefits in patients during long-term therapy with efalizumab and particularly in those of advanced age or with a history of immunosuppressive therapy.

In patients receiving any immunosuppressive therapy, dermatologists should be vigilant for signs and symptoms suggestive of PML. Suspicious cases should be referred to a neurologist.
